# Early lymphocyte levels and low doses radiation exposure of lung predict lymphopenia in radiotherapy for lung cancer

**DOI:** 10.3389/fimmu.2024.1426635

**Published:** 2024-08-01

**Authors:** Łukasz Kuncman, Matusz Pajdziński, Krzysztof Smółka, Mateusz Bilski, Joanna Socha, Rafał Stando, Magdalena Peszyńska-Piorun, Katarzyna Korab, Barbara Alicja Jereczek-Fossa, Jacek Fijuth

**Affiliations:** ^1^ Department of Radiotherapy, Medical University of Lodz, Lodz, Poland; ^2^ Department of External Beam Radiotherapy, Copernicus Memorial Hospital in Lodz Comprehensive Cancer Center and Traumatology, Lodz, Poland; ^3^ Institute of Mechatronics and Information Systems, Lodz University of Technology, Lodz, Poland; ^4^ Department of Radiotherapy, Medical University of Lublin, Lublin, Poland; ^5^ Department of Brachytherapy, Lublin Cancer Center, Lublin, Poland; ^6^ Department of Radiotherapy, Lublin Cancer Center, Lublin, Poland; ^7^ Department of Radiotherapy, Regional Oncology Center, Czestochowa, Poland; ^8^ Department of Radiation Oncology, Holycross Cancer Center, Kielce, Poland; ^9^ Radiotherapy Planning Department, Copernicus Memorial Hospital in Lodz Comprehensive Cancer Center and Traumatology, Lodz, Poland; ^10^ Department of Radiation Oncology, European Institute of Oncology IRCCS, Milan, Italy; ^11^ Department of Oncology and Hemato-Oncology, University of Milan, Milan, Italy

**Keywords:** lymphopenia, radiotherapy, immunotherapy, immune checkpoint inhibitors, radiation induced lymphopenia, effective dose to immune cells, lung cancer

## Abstract

**Introduction:**

Radiation induced lymphopenia (RIL) deteriorate survival and diminishes the benefit of immune checkpoint inhibitors in combined treatment of lung cancer. Given the inconsistent data across various studies on the predictors of RIL, we aim to methodically elucidate these predictors and formulate a practical guide for clinicians.

**Methods:**

We conducted observational cohort study in four tertiary cancer centers. Patients with non-small cell lung cancer and small cell lung cancer, without lymphopenia grade >1, who underwent standalone radiotherapy (RT) in minimum 15 fractions were eligible. Dose-volume parameters of structures and clinical factors were comprehensively analyzed using various predictors selection methods and statistical models (Linear Regressors, Elastic Net, Bayesian Regressors, Huber Regression, regression based on k-nearest neighbors, Gaussian Process Regressor, Decision Tree Regressor, Random Forest Regressor, eXtreme Gradient Boosting, Automated Machine Learning) and were ranked to predict lymphocytes count nadir (alc_nadir).

**Results:**

Two hundred thirty eight patients (stage I-3.4%, II-17.6%, III-75.2%, IV-3.8%) who underwent RT to median dose of 60 Gy were analyzed. Median alc_nadir was 0.68K/mm^3^. The 60 feature sets were evaluated in 600 models (RMSE 0.27-0.41K/mm^³^). The most important features were baseline lymphocyte count (alc_1), mean lung_dose, lung v05, lung v10, heart v05 and effective dose to immune cells (edic). In patients with alc_1 ≤ 2.005K/mm^3^, median alc_nadir predictions were 0.54K/mm3 for lung_v05p > 51.8% and 0.76K/mm^3^ for lung_v05p ≤ 51.8%. Lymphopenia was rare in patients with alc_1 > 2.005K/mm^3^.

**Discussion:**

RIL was most severe in patients with low early lymphocyte counts, primarily triggered by low RT doses in the heart and lungs.

## 1 Introduction

Landscape of treatment of lung cancer is evolving in recent years with increasing role of immunotherapy in treatment of all stages of non-small cell lung cancer (NSCLC) and small cell lung cancer (SCLC) (1–3). Immune checkpoint inhibitors (programmed cell death protein 1 (PD-1)/programmed death-ligand 1 (PD-L1) and cytotoxic T-lymphocyte-associated protein 4 (CTLA-4)) have become standard components in both the first line and subsequent lines of systemic therapy for metastatic NSCLC and SCLC (1, 3). Building on successes in metastatic cancer, those discoveries have been integrated into concurrent chemo-radiotherapy of advanced NSCLC, with results for SCLC anticipated in the coming years (4, 5). For patients ineligible for concurrent chemo-radiotherapy due to performance status, comorbidities, or loco-regional tumor burden, radiation therapy alone (RT) and sequential chemo-radiotherapy remain the alternatives, with ongoing trials also exploring the incorporation of immunotherapy in these scenarios (6, 7).

The incorporation of immune checkpoint inhibitors into the management of unresectable lung cancer has shifted the focus towards the immune system, especially highlighting the significance of lymphocytes. In the context of combined CRT and immune checkpoint inhibitors for treating unresectable, loco-regionally advanced lung cancer, the concern of lymphopenia becomes even more critical (8). RT is the primary culprit of lymphopenia due to the high radiosensitivity of lymphocytes, which are the most radiosensitive among all peripheral blood cells; this leads to radiation-induced lymphopenia (RIL) that impairs the effectiveness of consolidative immunotherapy (9–11). Additionally, treatment-related lymphopenia was found to reduce OS in lung cancer patients receiving immune checkpoint inhibitors (12).

Despite the compelling nature of the aforementioned data, unified guidelines for lymphocyte-sparing radiotherapy have yet to be established (13). Various studies present inconsistent data on how different dose-volume indicators influence treatment-related lymphopenia (11, 13). Two primary theories explain RIL: the first connects it to dose to immune cells in “blood rich” critical organs(heart, lungs, vessels) and the second associates it with bone marrow doses (14–19). Importantly, these theories, though separate, do not contradict each other in elucidating RIL. In numerous scientific publications, a variety of statistical models have been employed, complicating the comparison and evaluation of methods, and hindering their clinical implementation (13).

Our objective is to methodically evaluate the aforementioned hypotheses using diverse statistical modeling techniques across four distinct patient cohorts who underwent RT or sequential chemo-radiotherapy in tertiary cancer centers to find features which predict and potentially prevent RIL most accurate and create physician friendly algorithm.

## 2 Materials and methods

### 2.1 Study design and participants

In this retrospective multicenter observational cohort study, we included adult patients diagnosed with histopathological confirmed SCLC or NSCLC. Patients were eligible if they were qualified for standalone radical RT or sequential chemo-radiotherapybetween September 2019 and December 2022 by multidisciplinary board in four tertiary cancer centers in Poland. The RT was administered with curative intent with at least 15 fractions. Patients were excluded from the study if they presented with an absolute lymphocyte count (alc_1) of Common Terminology Criteria for Adverse Events (CTCAE) v5.0 grade >1 (less than 800 cells/cm^3^) at the initiation of RT or if they experienced a break in radiotherapy exceeding seven days for reasons not related to treatment complications. Data for this study were collected retrospectively from medical records and RT planning systems, while contours for the accessed target volume of total vertebral body bone marrow (vb) and the bone marrow volume (bm) were created prospectively for the purposes of the current study according to provided protocol. The vb area is defined as the volume of vertebrae body Th1-Th10. The bm volume encompasses all bones in the cranial-caudal dimension of Th1-Th10, including the vb area. All RT plans were recalculated after delineation of above structures.

Potential clinical and dosimetry predictors of RIL were derived based on the review of literature from the PubMed, Scopus, Web of Science, databases performed by ŁK and supervised by JF. Those factors were all in line with recently published LymphoTEC recommendations and metanalysis (8, 11). The data collected in accordance with the protocol included:Age in Years (age), Sex (female/male), Disease Stage (Stage; stages 1–4), Eastern Cooperative Oncology Group Performance Status (ECOG) (ecog_ps; 0–4), Prophilactic cranial irradiation before Lung RT (pci; No/Yes), absolute Lymphocyte Count from 1 Week of Radiotherapy (alc-1; in thousand/mm³), Nadir Absolute Lymphocyte Count during and up to Two Weeks Post-RT (alc_nadir; in thousand/mm³), Total Dose of Radiotherapy (rt_total_dose; in Gy), Fractional Dose of RT (rt_fraction_dose; in Gy), Duration of Radiotherapy (rt_duration; in days), Prior Chemotherapy (cht_before_rt; 0-No, 1-≤4 weeks, 2->4 weeks), Vertebral Body Volume (vb_v; in cm³), Mean Vertebral Body Dose Th1-Th10 (vb_md; in Gy), Percent Volume of Vertebral Body receiving 5, 10, 20, 30, and 40 Gy (vb_v05p, vb_v10p, vb_v20p, vb_v30p, vb_v40p), Volume of Bones (Bone Marrow) Th1-Th10 including Vertebral Body (bm_v; in cm³), Mean Dose in Bones Th1-Th10 including Vertebral Body (vb_md; in Gy), Percent Volume of Bone Marrow receiving 5, 10, 20, 30, and 40 Gy (bm_v05p, bm_v10p, bm_v20p, bm_v30p, bm_v40p), Volume of Planning Target Volume(ptv_v; in cm³), Heart Volume (heart_v; in cm³), Medium Heart Dose (heart_mhd; in Gy), Percent Volume of Heart receiving 5 and 10 Gy (heart_v05p, heart_v10p), Lung Volume (lung_v; in cm³), Medium Lung Dose (lung_mld; in Gy), Percent Volume of Lung receiving 5 and 10 Gy (lung_v05p, lung_v10p), Medium Body Dose Th1-Th10 (mbd; in Gy). The features calculated based on the collected data were: Absolute Lymphocyte Count Classification per CTCAE v5.0 (ctcae), Effective Dose to Immune Cells (edic) (15), RT-induced lymphopenia ≥G3, which was defined as Nadir Absolute Lymphocyte Count less than 0.5 (thousand/mm³). Full statistical description is provided in the **Supplementary Material**. Lymphocytes are extremely radiosensitive and as proposed in previous papers linear model (not linear-quadratic) was used for assessment dose-volume effect for primary endpoint (lymphocytes counts) (20–22). Additionally, this approach facilitates comparison of different dose-levels used for modeling.

The study adhered to ethical standards and received approval from the relevant institutional review boards/director of participating hospitals. According to the local regulation, the consent of the Bioethics Committee was not required due to the retrospective nature and anonymization of the data.

### 2.2 Primary endpoint

The primary endpoint of the study was to methodically determine the dose-volume parameter(s) in radiotherapy treatment plans and clinical factors that most accurately predict alc_nadir, across four independent lung cancer patient’s cohorts undergoing standalone RT or sequential chemo-radiotherapy.

### 2.3 Statistical analysis

**Figure 1:**
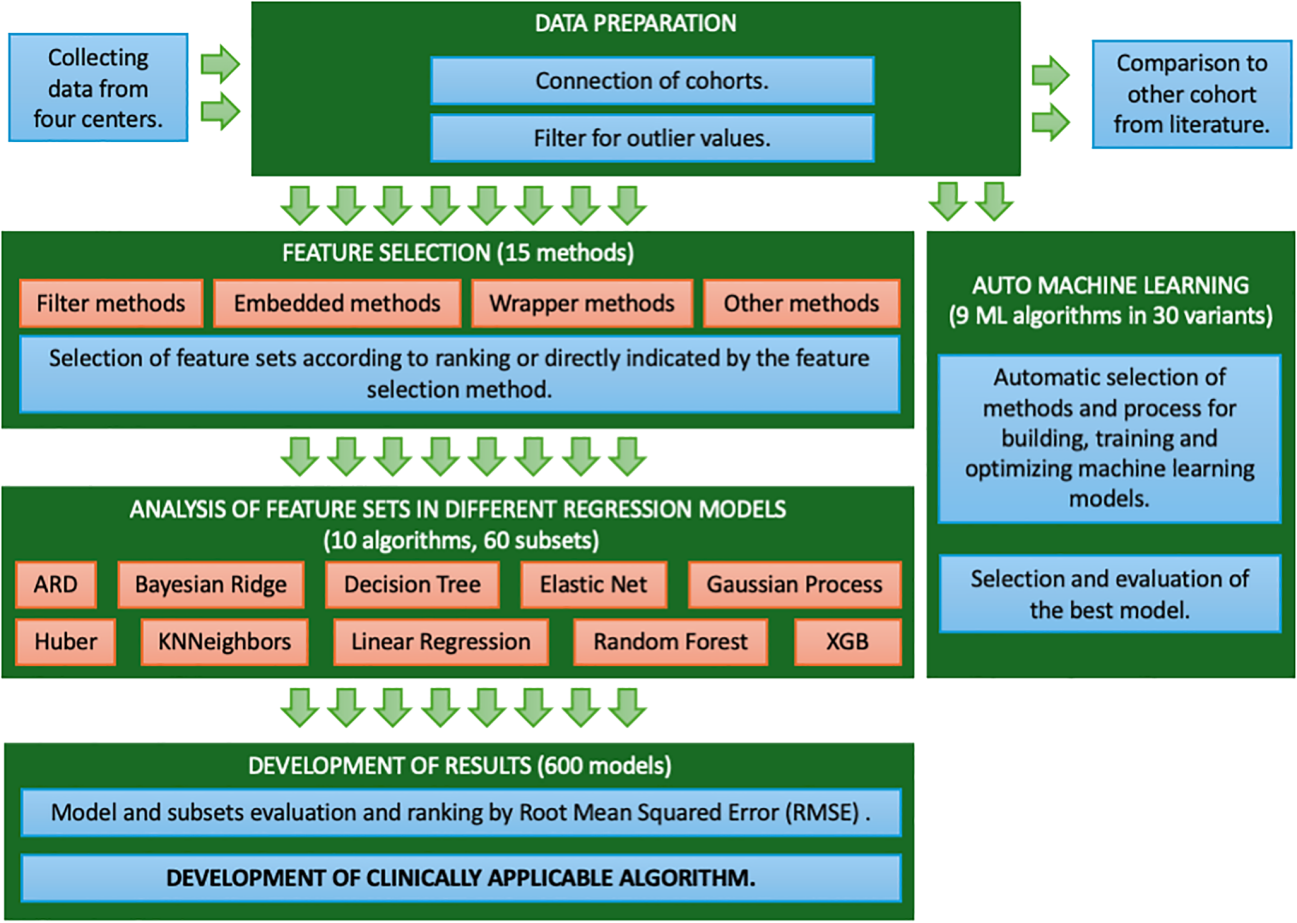
The study design. The figure shows workflow of study.

The study design is summarized in **Figure 1**. Briefly data was compiled from four tertial cancer centers, where rules based on boxplot procedures were used to remove outliers (the maximum alc_nadir value was set at 1.65 thousand/mm³, the maximum alc_1 value was set at 3.57 thousand/mm³). Of 258 patients, 238 were included to further analysis.

Firstly, we generated models for prediction of ≥3 CTCAE using commonly used algorithms (Random Forest, ADABost, XGBoost and Logistic Regression) to compare the dataset to the data in the existing literature. The Receiver Operating Characteristic (ROC) was used for analysis. Second step aimed to find dose-volume metrics and clinical feature which predicts alc-nadir with best accuracy. The continuous variable (alc_nadir) was chosen for evaluation as a more sensitive measure compared to categorical metrics (ctcae).

Subsequently, feature selection methods were applied, recognizing the significant impact of variable selection on model efficiency (23) Given the lack of the universal feature selection method, multiple diverse methods were utilized to identify key features (24, 25). The filter-based methods (Variance Threshold, Chi squared, ANOVA, Information gain, Correlation Coefficient, Fisher score, Information Value (IV)), embedded methods (Lasso Regularization, Random Forest Importance), wrapper methods (Forward Feature Selection, Backward Feature Elimination, Exhaustive Feature Selection, Recursive Feature Elimination, Recursive Feature Elimination with Cross Validation) and other methods (Shapley Values, hybrid methods, arbitrary features selection) as shown in **Figure 1**. Filter-based methods identify features by their statistical properties, such as Variance Threshold for low variance. Embedded methods like Lasso Regularization and Random Forest Importance select features during model training by shrinking coefficients or measuring feature impact on accuracy. Wrapper methods like Forward Feature Selection, Backward Feature Elimination, Exhaustive Feature Selection, Recursive Feature Elimination iteratively build and assess models to evaluate feature importance, using cross-validation for optimal feature selection. Other methods, such as Shapley Values, explain each feature’s contribution to predictions, while hybrid and arbitrary selection methods leverage multiple techniques and domain knowledge.

After the above-described selection, obtained feature sets were evaluated using ten different regression models, including classical Linear Regressors, Linear Regressor with variable selection (Elastic Net), Bayesian Regressors (Automatic Relevance Determination – ARD and Bayesian Ridge), Outlier-robust Regressor (Huber Regression), regression based on k-nearest neighbors (KNN), Gaussian Process Regressor (GPR), Decision Tree Regressor, ensemble methods (Random Forest Regressor and eXtreme Gradient Boosting - Xgboost). Five-group cross-validation was used to prevent overfitting and provide a stable and more reliable assessment. Obtained models were ranked based on Root Mean Square Error (RMSE). Parallel to the feature selection process, we optimized machine learning models using automated machine learning (AutoML) tools. In the model verification process for machine learning, the dataset was divided into three groups: training and validation data (collectively 75%) using five-fold cross-validation, and test data (25%). The training data were utilized for constructing and optimizing the model, the validation data facilitated the tuning of hyperparameters and prevention of overfitting through the selection of the optimal model configuration, while the test data enabled the assessment of the model’s final performance. After selection of features (max depth=2) decision tree was generated.

Calculations were performed using Python, scikit-learn (sklearn) machine learning (ML) library, dtreeviz library to visualized and interpreted decision trees, XGBoost Python package and statistical analysis package - statsmodels. An automatic machine learning library, AutoGluon, was used to optimize machine learning models. A Jupyter notebook was used as the integrated development environment (IDE).

## 3 Results

The 238 patients with NSCLC and SCLC (stage I-3.4%, II-17.6%, III-75.2%, IV-3.8%) in ECOG 0–3 performance status were enrolled. Patients were treated with hypo-fractionated RT in 62 cases and normo-fractionation in 176 cases to median RT dose of 60 Gy. Fraction doses ranged from1.8 to 3.0 Gy. Total duration of treatment was 19–76 days (median 40). Lymphopenia G1, G2, G3 and G4 occurred in 31.5%, 35.3%, 26.9%, 1.3% of patients, respectively. The median alc_nadir was 0.68K/mm^3^. Clinical data are summarized in **Table 1**. The description statistics of all collected dose-volume and clinical data are summarized in **Supplementary Table S1** in **Supplementary Material**.

**Table 1:** Patient characteristics.

Feature	Total	CEN 1	CEN 2	CEN 3	CEN 4
**Patients**	238	141	25	34	38
Sex					
female	93	57	8	15	13
male	145	84	17	19	25
Age					
min	39	50	39	54	45
max	85	85	84	85	76
median	67	67	70	67	67
Disease stage (I-IV)					
I	8	4	1	3	0
II	42	19	5	6	12
III	179	115	18	20	26
IV	9	3	1	5	0
ALC from 1 week RT (thousand/mm³)					
min	0.81	0.81	0.94	1.10	0.81
max	3.43	3.43	3.00	3.38	3.24
median	1.76	1.63	1.97	1.97	1.72
ALC nadir (thousand/mm³)					
min	0.15	0.15	0.27	0.18	0.19
max	1.58	1.51	1.58	1.56	1.53
median	0.68	0.66	1.13	0.58	0.68
Total RT dose (Gy)					
min	40	40	42	53	56
max	68	66	66	68	66
median	60	60	60	60	66
Hypofractionation (YES: RT_fraction_dose>2, NO: RT_fraction_dose ≤ 2)					
YES	62	34	12	12	4
NO	176	107	13	22	34
V PTV - Volume planning target (cm³)					
min	55	55	70	66	105
max	1149	1058	860	1149	666
median	292	257	452	360	372
Lymphopenia CTCAE v5.0					
G0	12	4	4	2	2
G1	75	40	15	11	9
G2	84	58	2	8	16
G3	64	38	4	12	10
G4	3	1	0	1	1
G5	0	0	0	0	0
Chemotherapy before RT?					
No	73	46	6	8	13
CHT ≤ 4 weeks	36	0	11	13	12
CHT>4 weeks	129	95	8	13	13

**Figure 2:**
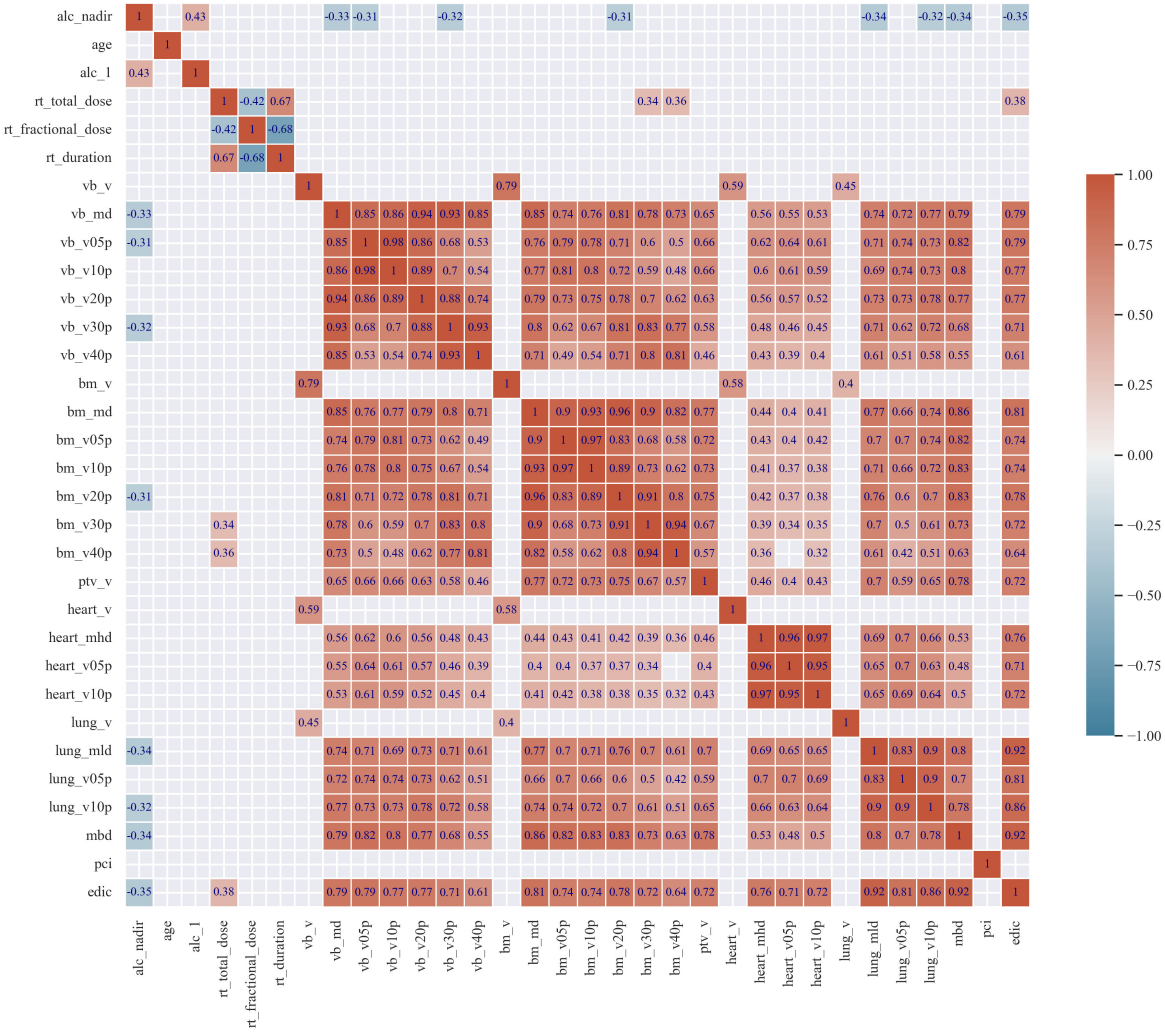
The correlation matrix. The correlation matrix heatmap for the values of the Spearman correlation coefficients (r) for analyzed features. For clarity of the correlogram, only coefficients (r) greater than 0.3 are shown.

As anticipated, we identified significant positive correlations among dose-volume parameters across distinct categories, specifically within regions including the heart (heart_mhd, heart_v05, heart_v10), lungs (lung_mld, lung_v05, lung_v10), bone marrow (bm_md, bm_v05, bm_v10, bm_v20, bm_v30, bm_v40), and vertebral bodies (vb_md, vb_v05, vb_v10, vb_v20, vb_v30, vb_v40), as delineated in the correlation matrix presented in **Figure 2**. The variables within these groups exhibited strong collinearity.

**Figure 3:**
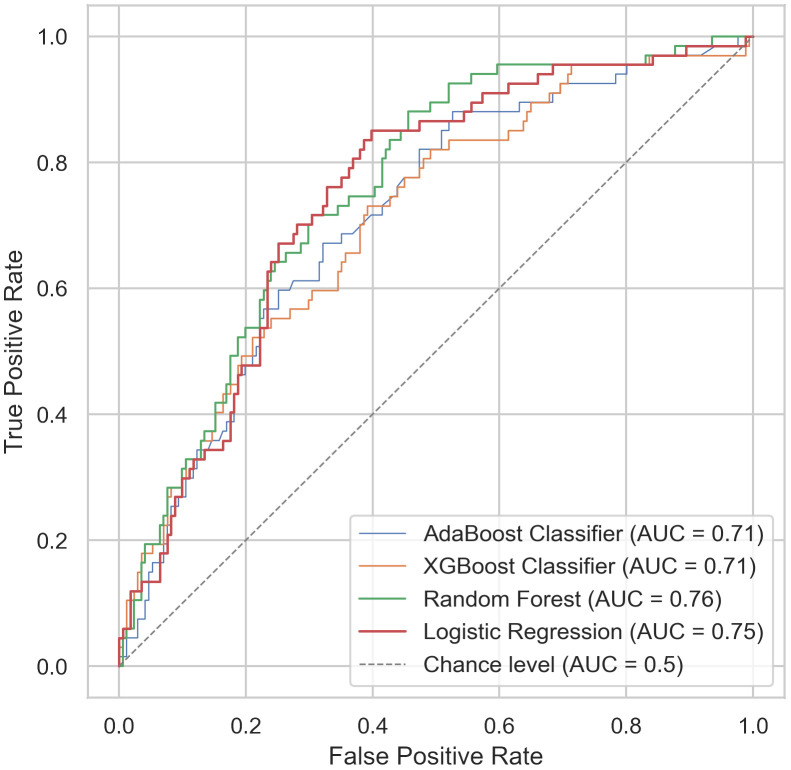
Prediction of Common Terminology Criteria for Adverse Events (CTCAE) ≥ grade 3 lymphopenia. Receiver Operator Curves for prediction of Common Terminology Criteria for Adverse Events (CTCAE) ≥ grade 3 lymphopenia.

According to the calculations performed using four classifiers (AdaBoost, XGBoost, Random Forest, Logistic Regression), shown on **Figure 3**, models yield good performance in prediction of ctcae with area under curve (AUC) ranging from 0.71 to 0.76, with best performance for Random Forest and Logistic Regression. The base details of model were as follows: 5 features (alc_1, lung_mld, lung_v05p, heart_v05p, sex) and 5 fold cross validation. The variance in the ROC metric incorporating individual results from cross-validation analyzes of best model was depicted in **Supplementary Figure S1** in **Supplementary Material**. Our models had comparable performance in prediction of ctcae toxicity to those described in the literature thus we moved further to the next step described in **Figure 1** (26–29).

**Figure 4:**
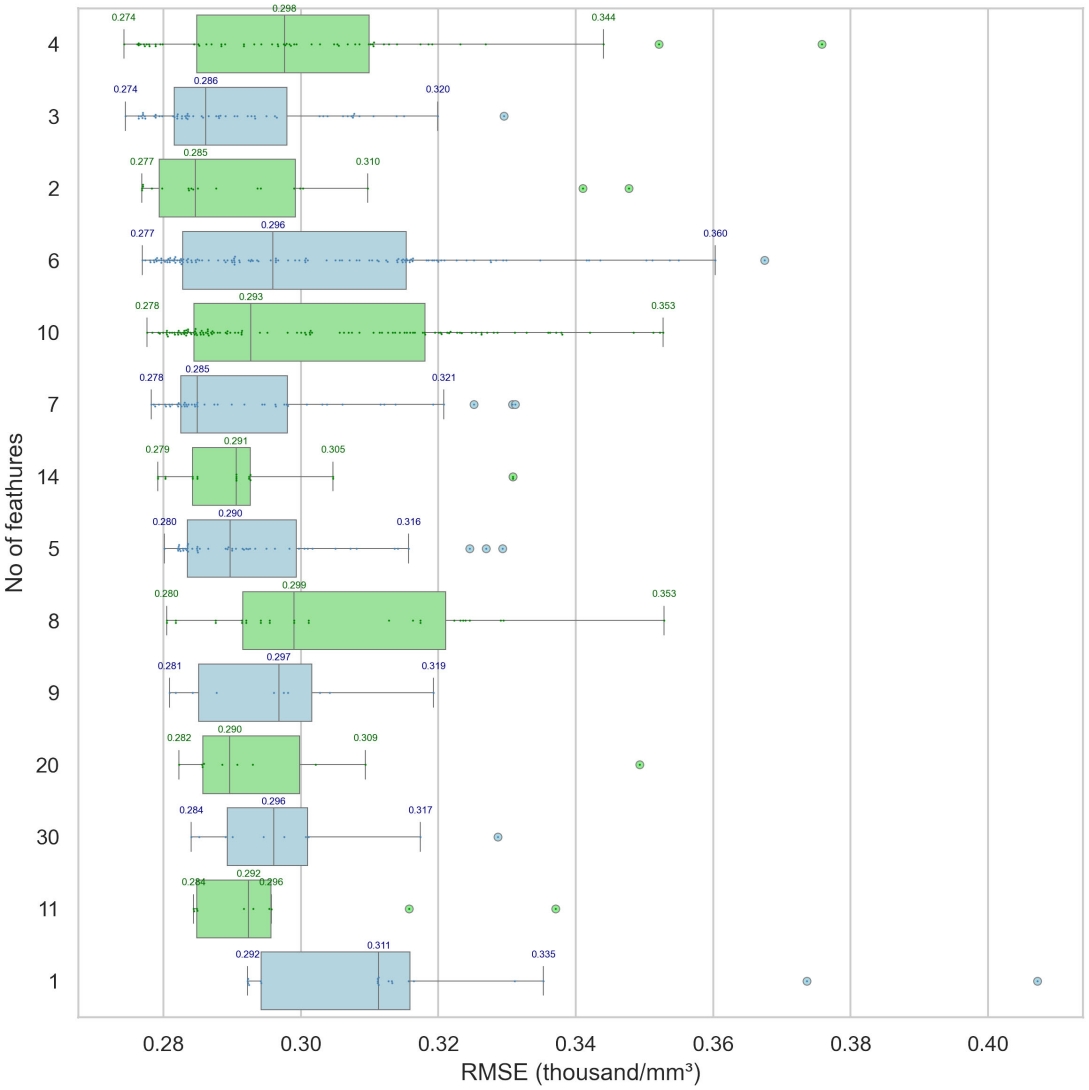
The optimal outcome, as indicated by the lowest Root Mean Square Error (RMSE) of individual models depending on the number of selected features. The models with specific number of features are grouped according to increasing RMSE. The dots represent the actual RMSE values of the models. The boxplots of the root mean square error (RMSE) values for individual models are displayed along the X-axis, as a function of the number of selected features, which are indicated on the Y-axis. The plots are organized in ascending order of the minimum RMSE value. Models incorporating fewer features proved to be the most effective; notably, the set containing three variables was an optimal selection across all computational models assessed.

**Figure 5:**
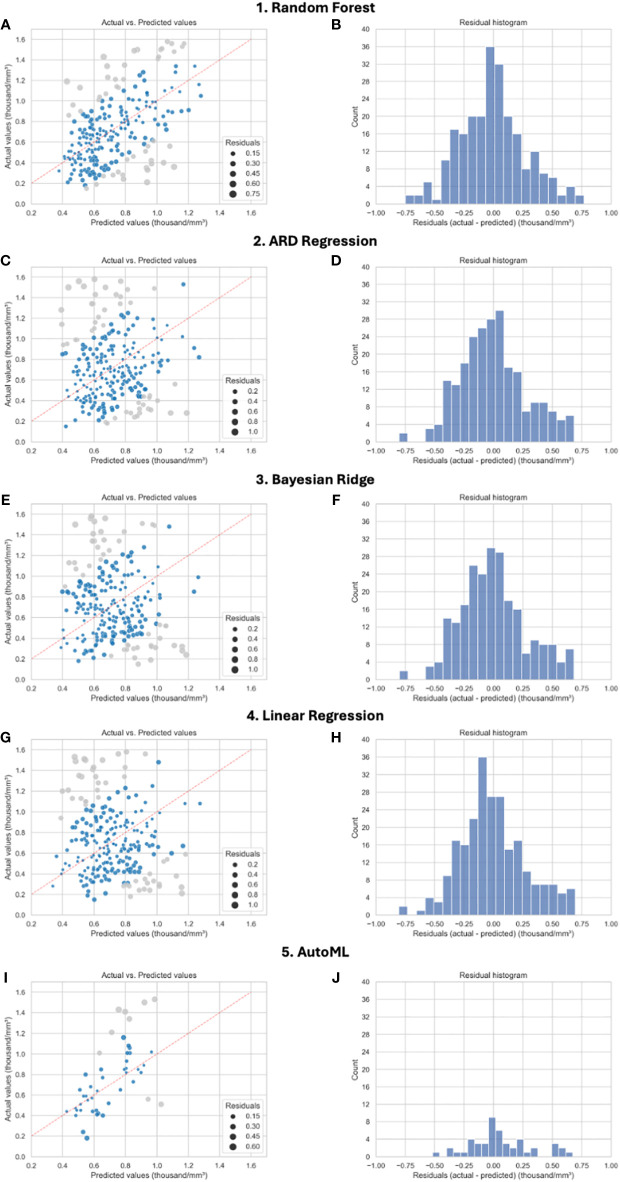
**(A–J)** Model performance in prediction absolute lymphocyte count (alc_nadir). Comparison of most efficient models for prediction alc_nadir. The plots on the left show the actual values vs predicted values given by the models. The darker color applies to the top 80% of results. A perfect regression model would display data points on the diagonal defined by predicted equal to actual values. The size of the points is related to the residuals. The plots on the right show residual histograms. For automatic machine learning (AutoML) the values come only from the test set (25%). The split is as follows: 75% of the set was used for training data, on which 5-fold cross-validation was again applied. This set therefore contains both training and validation data. The results testing was based on the remaining 25% of the data set. For all other models, cross-validation with 5-fold splits was used (all data were used for both training and testing). The final result is based on the average value obtained from the individual sets.

Feature selection revealed 60 feature sets which were evaluated using ten different regression models and yield 600 models of alc_nadir prediction. Models consisting of a larger number of features did not demonstrate greater efficacy as shown in **Figure 4**. The detailed comparison of individual and grouped feature selection methods, as well as the performance of the corresponding models, is presented in **Supplementary Figure S2**. The **Table 2** displays the efficacy of the five leading types of models (best one from each type) according to Root Mean Squared Error (RMSE), best 30 models are shown in **Supplementary Table S2** in **Supplementary Material**. The RMSE and accuracy ranged 0.27–0.41 thousand/mm³ and 47–62% respectively. Leading models presented similar efficacy (best three were Random Forest Regressor as shown in **Supplementary Table S2**). The scatter plots and histograms of themodels from **Table 2** are shown in **Figures 5A–J** and represents a comparison between actual and predicted values. The clustering of points along the dashed line suggests a good model fit, especially for AutoML model. The most important features consistently observed across the models were: alc_1, lung_mld, lung_v05p, lung_v10p, heart_v05p and edic (calculated from lung_mld, heart_mld, mbd, number of fractions).

**Table 2:** Best performing models for prediction of absolute lymphocyte count nadir.

Id	Method	Features	No of features	MAE (thousand/mm³)	RMSE (thousand/mm³)	Accuracy (%)
1	**Random Forest** cross validation, 5 fold	alc_1, heart_v05p, lung_mld, lung_v05p	4	0.213	0.274	60.58
2	**ARD Regression** cross validation, 5 fold	alc_1, lung_mld, lung_v05p	3	0.219	0.276	60.01
3	**Bayesian Ridge** cross validation, 5 fold	alc_1, lung_mld, lung_v05p, lung_v10p	4	0.219	0.277	60.13
4	**Linear Regression** cross validation, 5 fold	alc_1, edic	2 (5)	0.218	0.277	60.33
5	**AutoML** train (75%): cross validation, 5 fold, test (25%)	All	30	0.195 (validation) 0.201 (test score)	0.252 (validation) 0.272 (test score)	70.15 (test score)

Models selected according to Root Mean Squared Error (RMSE) and automatic machine learning method prediction is presented. RMSE, Mean Absolute Error (MAE), Accuracy =100%−MAPE, where MAPE – Mean Absolute Percentage Error, also known as mean absolute percentage deviation (MAPD) is shown.

**Figure 6:**
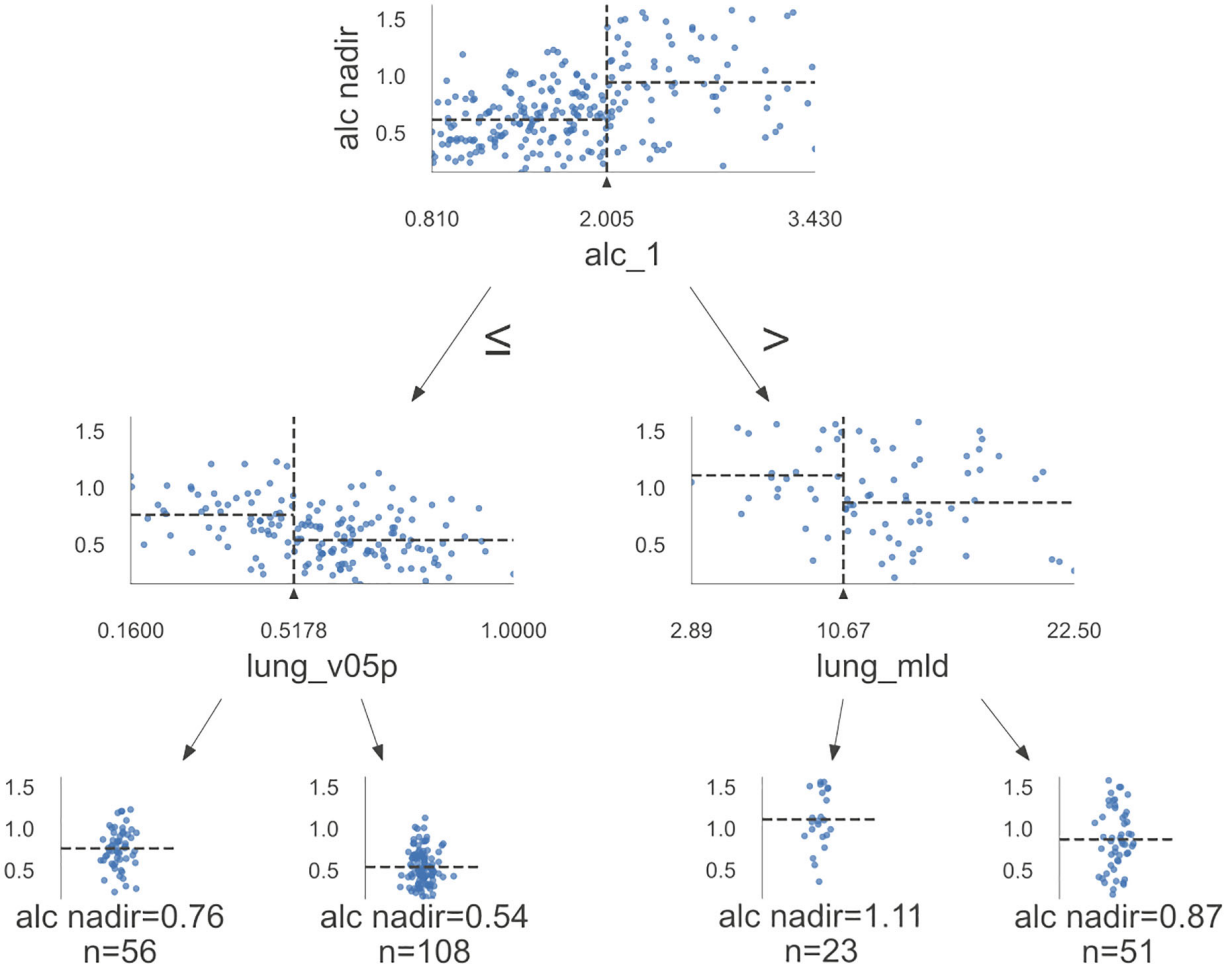
Algorithm for prediction of absolute lymphocyte count nadir (alc_nadir). Decision tree model with decision making path, max depth=2, [alc_1, heart_v05p, lung_v5p, lung_v10p, lung_mld] RMSE = 0.29.

Final step aimed to generate clinically applicable algorithm to guide physicians. Decision tree from variables from best performing models included in **Table 2** is shown on **Figure 6**. The alc_nadir was predicted based on alc_1, lung_v05p, and lung_mld. The decision tree model (RMSE = 0.29) identified alc_1 of 2.005K/mm^3^ as the threshold in the first step of selection. For patients with alc_1 values less than or equal to 2.005K/mm^3^ and lung_v05p ≤ 51.8%, a median alc_nadir of 0.76K/mm^3^ was estimated, while for those with lung_v05p > 51.8%, a median alc_nadir of 0.54K/mm^3^ was predicted. For patients with alc_1 greater than 2.005K/mm^3^, a lung_mld threshold of 10.67Gy segregated patients into groups with predicted median alc_nadir values of 1.11K/mm^3^and 0.87K/mm^3^.

## 4 Discussion

Lymphocytes are the most radiosensitive blood cells, and although often underreported, lymphopenia is the most prevalent hematologic toxicity, with grade ≥ 2 and ≥3 occurring in our cohort in 63% and 28% of cases respectively (8). Lymphopenia is widely recognized as an unfavorable prognostic factor for both progression-free survival (PFS) and overall survival (OS) in patients with various cancers including NSCLC and SCLC (30, 31). Similarly, RIL has been shown to negatively impact OS and PFS in NSCLC and SCLC patients (8, 11, 13, 31). It’s important to note that most studies linking RIL with lower survival were done before immunotherapy was used for lung cancer. The introduction immune checkpoint inhibitors might further amplify this negative impact, especially given that lymphopenia induced by treatment is a recognized as unfavorable prognostic factor for both OS and PFS during immune checkpoint inhibitors therapy (12). Indeed, severe RIL diminishes the survival advantages of durvalumab following concurrent chemo-radiotherapy in NSCLC as shown recently (9). Additionally, the Real World Evidence (RWE) studies confirm efficacy of consolidative immune checkpoint inhibitors after CRT in population with more elderly and poor performance status patients and may double overall survival in that population (32, 33). Therefore, developing strategies to minimize RIL could be crucial in improving the effectiveness of comprehensive lung cancer treatment approaches as never before.

Our analysis showed that from non-modifiable factors lymphocyte count at the beginning of treatment (alc_1) was predictive on occurrence of lymphocyte nadir (alc_nadir) further during treatment which stays in line with some of previously published papers (13, 34, 35). Moreover, in our analysis, alc_1 proved to be the main factor, appearing in all top 30 models. Based on our analysis in patients with an alc_1 > 2.005K/mm^3^, clinically significant lymphopenia should not be anticipated. The value of our analysis is enhanced by excluding patients with lymphopenia (often caused by previous treatment).

In terms of clinical application, controlling the RT dose to critical structures like the lungs and a heart offer an opportunity to modify risk factors and reduce risk of RIL what is possible with use of modern irradiation techniques(including protons) (8, 36). This adjustment could lead to a reduction in RIL, especially valuable for patients with non-modifiable risk factors for lymphopenia, such as advanced age, lower pre-RT lymphocyte count, and larger tumor size (12). Considering the immune system as an organ-at-risk (OAR) in RT planning is complex, as it is not confined to a specific anatomical area (37). Immune cells circulate throughout the body, often moving in and out of the RT field, which challenges traditional RT planning approaches. From clinical point of view, there are two prevailing theories regarding the negative impact of RT on the count of immune system cells. The first concept emphasizes the significant role of the dose in the circulating pool of immune cells with mostly used edic model in causing RIL, and it’s directly related to the dose in critical organs such as the lungs, heart, and median body dose (14–17, 38). The second theory focuses on the impact of the dose in bone marrow on hematologic toxicity (18, 19). These two theories are not mutually exclusive but are rarely accessed together in studies; high doses in the lungs and heart strongly correlate with the dose in the chest bones, as evidenced by our correlation matrix.

Despite testing numerous sets of clinical and diametric features in our cohort of patients, most significant models demonstrated similar performance in prediction of alc_nadir. Reassuringly, in the era of artificial intelligence and advanced statistical methods, the subjective selection of features made by experts (heuristic methods) based on their experience and knowledge exhibited the best fit, as shown in **Supplementary Figure S2**. However, it also showed high variability. This variability may arise because experts can make mistakes or simply not choose optimal solutions. Although expert knowledge is often undervalued in modern science, our study indicates that combining statistical methods with expert knowledge can yield the best results. Surprisingly, three-five variables models presented best performance in which lung_mld, lung_v05p, lung_v10p, heart_v05p or edic played crucial role in prediction RIL. It’s worth to emphasize that low doses variables (lung_v05p, lung_v10p, heart_v05p) shows a strong correlation with lung_mld and heart_mld and edic. Similar results concerning heart and lung dose metrics were observed in study by Abravan et al., where data mining techniques were employed to discern regions where a significant correlation exists between RT dose and ≥G3 lymphopenia (31). Heart, lungs, and thoracic vertebrae were identified as regions linked to RIL, with key diametric parameters being mean doses to the lungs and heart and thoracic vertebrae V20 (31). In other studies, Tang et al. (lung_v05, lung_v10) and Xie et al. (lung_v05) and Kim et al. (lung_v05) and Kong et al. (lung_v05) observed that low doses in the lung were associated with RIL (39–42). Additionally, studies have demonstrated that low doses to the heart (heart_v05) are crucial in the induction of RIL during stereotactic body radiation therapy (SBRT) and CRT (43, 44). The metrics edic and EDRIC (Estimated Dose of Radiation to the Immune System) were also predictive of RIL (14, 16). Similarly, Kim et al. noted that the dose to circulating blood cells (as a function of lung and heart dose) was associated with severe RIL during photon and proton radiotherapy (36). Other studies addressing various malignancies in thoracic region have identified relationship between mean and low doses in heart and lung (and low doses) and RIL (11, 26). Our analysis showed that the threshold values of lung_v05p ≤ 51.8% and lung_mld of 10.67Gy can are significant in prediction of alc_nadir for patients with alc_1 ≤2.005K/mm^3^ and alc_1 >2.005K/mm^3^ respectively.

In our most effective models, the features previously outlined in literature such as age and tumor volume, treatment time, hypofractionation and pci usage were absent; however, they were incorporated in subsequent(less efficient) models (39, 40, 44). Surprisingly, previously described correlation of low-medium dose metrics of vertebrae(v05-v20) and other of bones with hematologic toxicity have been rarely observed in our best models (18, 19, 31, 45). It is noteworthy that dose metrics for vertebrae and other bones can serve as proxies for lung and heart dose metrics, as demonstrated in our correlation matrix. Furthermore, in some of the aforementioned studies where bones doses were associated with RIL, heart and lung doses were not assessed which complicate comparison (18, 19, 45). Additionally, it may be assumed that since approximately 50% of active bone marrow is in the pelvis, the dose metrics of bone marrow in this region may be critical in the induction of RIL. This correlation has been observed in numerous studies focusing on gynecological, genitourinary, and lower gastrointestinal malignancies (11, 35). Another explanation for the lack of impact of the dose-volume parameters of vertebrae and bones on lymphopenia could be the bone marrow regeneration observed during irradiation, which was assessed in patients undergoing chemoradiotherapy of the pelvic area (46).

What is important is that different validation techniques are used in the analyzed statistics methods. Only automated machine learning involves a split into training and test sets. The small number of patients is a limitation, particularly for the interpretability of machine learning models. Training on small datasets can lead to greater variability, making predictions less stable and more sensitive to input data changes. In our study, we used cross-validation techniques to reduce overfitting and enhance result reliability. We used various statistical methods to optimally select features affecting lymphopenia, which had not been done before. The limitation of our study arises from its retrospective nature and the relatively small, yet considerable, number of patients, given the stringent inclusion criteria (no initial lymphopenia, standalone RT treatment, and RT delivered in at least 15 fractions). These criteria resulted in a cohort comprised primarily of fragile patients, which does not fully represent the broader population eligible for concurrent chemo-radiotherapy with consolidative immune checkpoint inhibitors. Our analysis did not summarize late lymphopenia due to the difficulty in its assessment in a retrospective study (irregularity of blood parameter measurements post-chemoradiotherapy), which may constitute a limitation of the study.

The strength of our multicenter study is that we focused specifically on the patients undergoing RT alone, without concurrent systemic treatment and without lymphopenia at the beginning of treatment. This approach aimed to minimize the influence of chemotherapy on study endpoint as patient who suffered from lymphopenia before RT were excluded. We selected the more sensitive numerical variable, alc_nadir, over ctcae toxicity, and subsequently conducted a comprehensive testing of various statistical models and variable selection strategies, generating 600 predictive models—a scope of analysis that, to the best of our knowledge, has never been done so comprehensively before.

## 5 Conclusions

The RIL was predominantly triggered by low and median RT doses in the heart and lungs (e.g., lung_mld, lung_v05p, lung_v10p, heart_v05p or edic), with lesser impact from bone marrow-dependent doses. RIL was most severe in patients who had lower initial lymphocyte counts. For patients with alc_1 values less or equal than 2.005/mm^3^ and lung_v05p ≤ 51.8%, a median alc_nadir of 0.76K/mm^3^ was estimated, whereas for those with lung_v05p > 51.8%, a median alc_nadir of 0.54K/mm^3^was estimated.

## Data availability statement

The datasets presented in this study can be found in online repositories. The names of the repository/repositories and accession number(s) can be found below: https://1drv.ms/f/s!AqwHYmZlPESTg51KmmQc6Vo0OppBmQ?e=dBmAc5.

## Ethics statement

Ethical approval was not required for the study involving humans in accordance with the local legislation and institutional requirements. Written informed consent to participate in this study was not required from the participants or the participants’ legal guardians/next of kin in accordance with the national legislation and the institutional requirements.

## Author contributions

ŁK: Conceptualization, Formal Analysis, Methodology, Project administration, Supervision, Validation, Writing – original draft, Writing – review & editing. MP: Investigation, Resources, Writing – review & editing. KS: Data curation, Formal Analysis, Investigation, Methodology, Software, Visualization, Writing – review & editing. MB: Investigation, Resources, Writing – review & editing. JS: Investigation, Resources, Writing – review & editing. RS: Investigation, Resources, Writing – review & editing. MP-P: Investigation, Resources, Writing – review & editing. KK: Investigation, Resources, Writing – review & editing. BJ-F: Supervision, Writing – review & editing. JF: Supervision, Writing – review & editing.
