# Supplementary material for: Early lymphocyte levels and low doses radiation exposure of lung predict lymphopenia in radiotherapy for lung cancer
Source: Front Immunol. 2024 Aug 1;15:1426635. doi: 10.3389/fimmu.2024.1426635 (PMC11324483; doi:10.3389/fimmu.2024.1426635)
Supplement: Supplementary file 1 [file DataSheet_1.docx]

Supplementary Material

Radiation-Induced Lymphopenia Depend on Lung/Heart Irradiation Doses: A Comprehensive Comparative Study of Various Models Across Four Independent Patient Cohorts

Łukasz Kuncman, Matusz Pajdziński, Krzysztof Smółka, Mateusz Bilski, Rafał Stando, Joanna Socha, Magdalena Peszyńska-Piorun, Katarzyna Korab, Barbara Alicja Jereczek-Fossa, Jacek Fijuth

# Supplementary Figures and Tables

## Supplementary Figures

**Classification and ROC analysis**

Model for prediction of ≥3 Common Terminology Criteria for Adverse Events using Random Forest classification with cross-validation and base on several selected features (sex, absolute Lymphocyte Count from 1stWeek of Radiotherapy, Percent Volume of Heart receiving 5 and 10 Gray, Percent Volume of Lung receiving 5 Gray and Prior Chemotherapy) were generated to compare the dataset to data in existing literature.


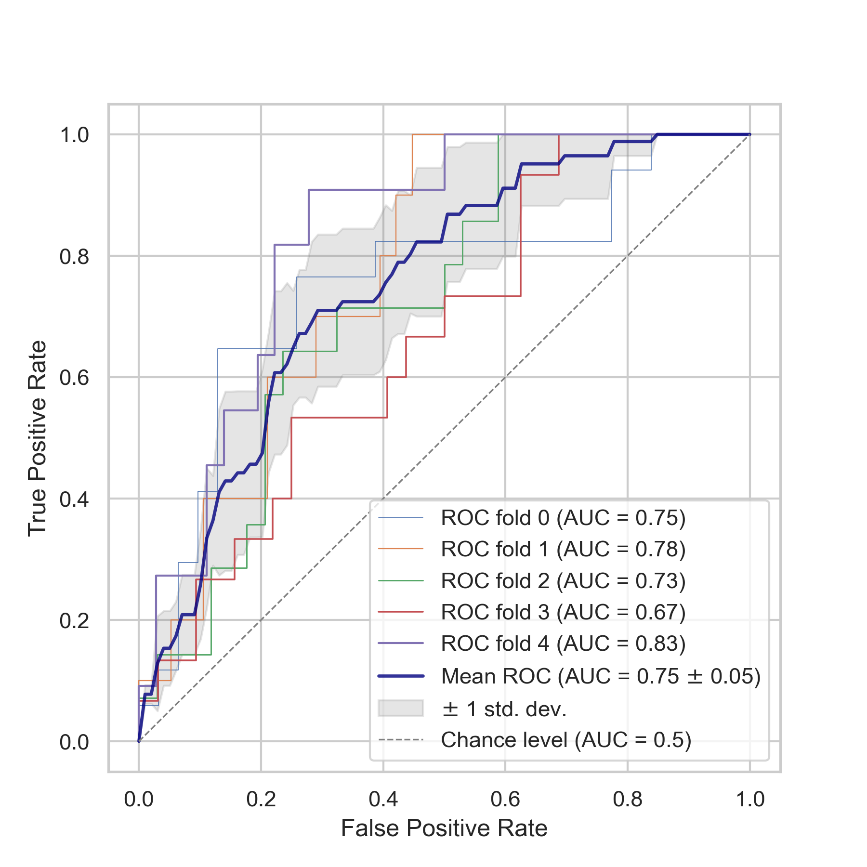

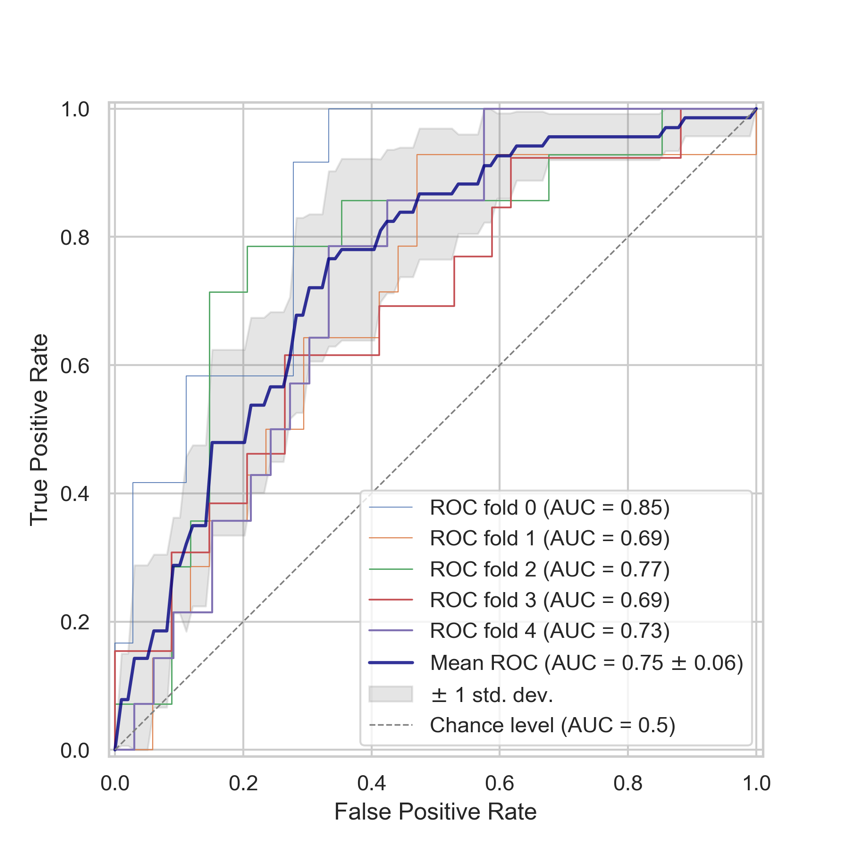


a

b

**Supplementary Figure S1.** The Receiver Operating Characteristic (ROC) for model for prediction of ≥3 Common Terminology Criteria for Adverse Events using Random Forest classification on left (Figure S1a) and Logistic Regression classification on right (Figure S1b) and several selected features

The Figure S2 shows the ROC response of different datasets, created from K-fold cross-validation. Taking all of these curves, it is possible to calculate the mean AUC, and see the variance of the curve when the training set is split into different subsets. This shows how the classifier output is affected by changes in the training data, and how different the splits generated by K-fold cross-validation are from one another.

The results for individual subsets and the average AUC value do not differ from those found in the literature.


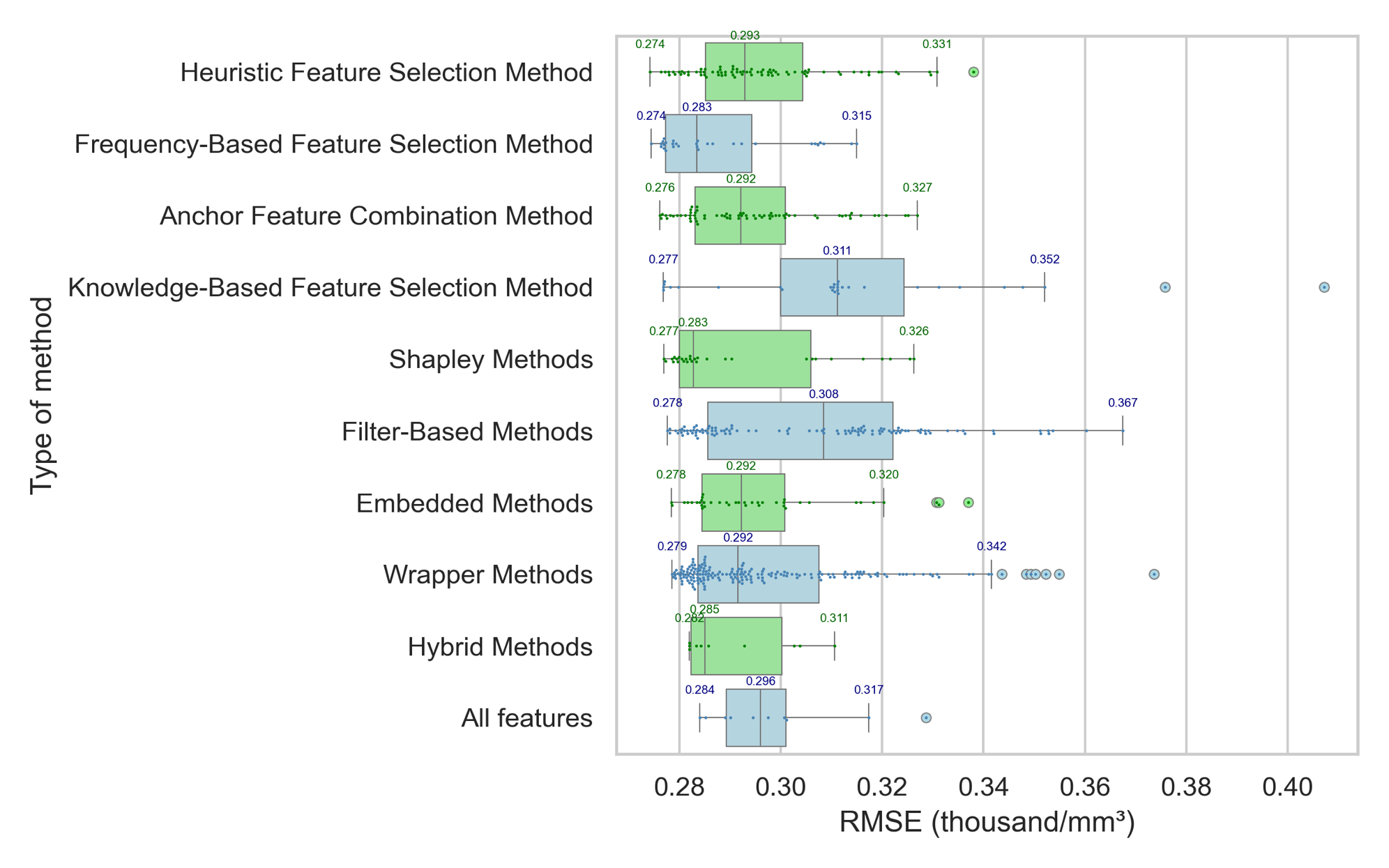


**Supplementary Figure S2.** The optimal outcome, as indicated by the lowest Root Mean Square Error (RMSE) of individual models depending on the type of feature selection method. The methods are grouped according to increasing RMSE. The dots represent the actual RMSE values of the models.

The following division of feature selection methods was used:

**Heuristic Feature Selection Method** – method where selections are based on general assumptions or simple rules that are not strictly defined, often involving domain-specific knowledge. This approach, albeit somewhat arbitrary, sometimes yields notable results. Set of features selected by experts (authors) were as follows: 1)alc_1, heart_v05p, lung_mld, lung_v05p, 2)alc_1, cht_before_rt, disease_stage, ecog_ps, heart_mhd, 3)ung_v10p, rt_total_dose, 4)alc_1, ecog_ps, lung_v05p, lung_v10p, ptv_v, 5)alc_1, heart_v05p, sex, vb_v20p, 5) alc_1, ecog_ps, ptv_v, 6)alc_1, lung_v10p, mbd, rt_duration, rt_fractional_dose, vb_md, vb_v10p, vb_v20p, 7)alc_1, heart_v05p, lung_v05p, lung_v10p, mbd, rt_duration, vb_md, vb_v10p, vb_v20p, 8)alc_1, bm_v20p, cht_before_rt, disease_stage, heart_mhd, heart_v05p, heart_v10p, sex, vb_v10p, vb_v20p
**Frequency-Based Feature Selection Method** - method where features are chosen based on their frequency of appearing as significant across various selection methods.
**Anchor Feature Combination Method** - method suggesting that one key feature ('anchor') is combined with other features to explore their collective properties or impact. In this case, it involved combining alc_1, as the most significant feature, with others.
**Knowledge-Based Feature Selection Method** - method where feature selection decisions are guided by previously acquired knowledge or evidence of their utility. In our case, these are sets based on the EDIC value or the features from which EDIC is derived. As observed, the average performance is the poorest, although it is possible to select a method that yields a fairly good result.
**Shapley Methods** – this method, often indicated (hence its separate categorization, rather than as a hybrid), determines the importance of features based on the constructed model. In our experience, much depends on the chosen model, and it did not perform optimally in our case.

**Filter-Based Methods** - feature selection methods that involve using statistical measures to assess and rank the importance of each feature in relation to the target variable.

**Embedded Methods** - methods that integrate the feature selection process within the training of the machine learning model itself, using model-specific algorithms to both assess the importance of each feature and simultaneously optimize the model.

**Wrapped methods** – methods that iteratively evaluate subsets of features, using a specific machine learning model as the evaluation criterion.

**Hybrid methods** - methods that use other feature selection methods together.

**All features** – indicating no selection was applied.

The optimal outcome, as indicated by the lowest root mean square error (RMSE), was attained using a method that relied on the domain-specific selection of features by an expert. However, this approach did not consistently yield the best feature sets and lacks automation. Consequently, a more effective method appears to be one that aggregates features most frequently identified as significant across various feature selection methods, primarily those based on filtering. Typically, single filter-based methods alone did not achieve comparably favorable results.


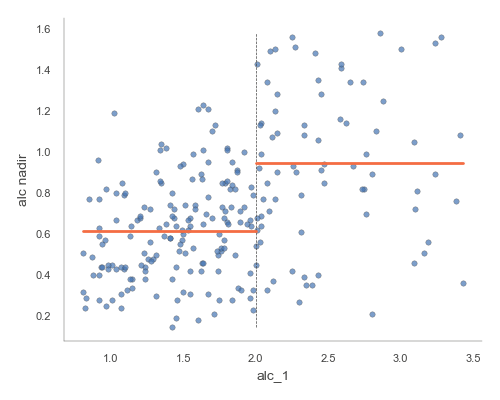


**Supplementary Figure S3**

The figure shows how the decision tree divides alc_1 to obtain the target alc_nadir values.


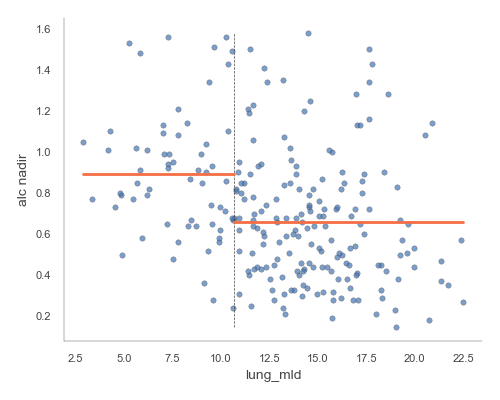


**Supplementary Figure S4**

The figure shows how the decision tree divides lung_mld to obtain the target alc_nadir values.

## Supplementary Tables

**Description statistics of collected data.**

| **Feature** | **Label** | **Type** | **Description** |
| --- | --- | --- | --- |
| **idglobal** | **id** | **skip** |  |
| Source | source | **categorical** | CEN1(149)/CEN2(33)/CEN3(35)/CEN4(41) |
| id | id | **skip** |  |
| Age (years) | age | **numerical** | min=39 max=85 median=67 mean=67.31 std=7.05 |
| Sex (female/male) | sex | **categorical** | MALE(154)/FEMALE(104) |
| Disease stage (1 - 4) | disease_stage | **categorical** | 1(9)/2(45)/3(194)/4(10)/5(0) |
| ECOG-PS (0-5) | ecog_ps | **categorical** | 0(12)/1(179)/2(62)/3(5)/4(0) |
| PCI (before lung: no/yes) | pci | **categorical** | YES(38)/NO(220) |
| ALC from 1 week of RT (thousand/mm³) | alc_1 | **numerical** | min=0.81 max=6.26 median=1.78 mean=1.93 std=0.85 |
| ALC nadir, the lowest ALC value during RT and up to two weeks after RT (thousand/mm³) (max 6) | alc_nadir | **numerical** | min=0.1 max=4.9 median=0.69 mean=0.78 std=0.51 |
| Total dose RT (Gy) (max 80) | RT_total_dose | **numerical** | min=40 max=68 median=60 mean=59.93 std=5.2 |
| Fractional dose RT (Gy) | RT_fractional_dose | **numerical** | min=1.8 max=3 median=2 mean=2.19 std=0.34 |
| RT duration (days) | RT_duration | **numerical** | min=19 max=76 median=40 mean=38.22 std=8.47 |
| Chemotherapy (CHT) before RT? - if "no" enter "0"; - if "yes" then:   - enter 1 - CHT≤4 weeks   - enter 2 - CHT>4 weeks | cht_before_rt | **categorical** | 0(78)/1(40)/2(140) |
| V TVB  Total vertebral body volume (cm³) | vb_v | **numerical** | min=113.5 max=354.78 median=193.56 mean=194.65 std=45.79 |
| MeanD TVB Mean total vertebral body dose Th1-Th10 (Gy) | vb_md | **numerical** | min=2.13 max=38.3 median=15.35 mean=16.03 std=6.72 |
| V5TVB (%) | vb_v05p | **numerical** | min=0 max=1.29 median=0.51 mean=0.53 std=0.16 |
| V10TVB (%) | vb_v10p | **numerical** | min=0 max=0.91 median=0.46 mean=0.47 std=0.16 |
| V20TVB (%) | vb_v20p | **numerical** | min=0 max=2.33 median=0.37 mean=0.37 std=0.21 |
| V30TVB (%) | vb_v30p | **numerical** | min=0 max=0.71 median=0.24 mean=0.25 std=0.16 |
| V40TVB (%) | vb_v40p | **numerical** | min=0 max=0.49 median=0.09 mean=0.13 std=0.12 |
| Volume of bones Th1-Th10 inc. TVB, V BM (cm³) | bm_v | **numerical** | min=337.25 max=2251.6 median=1251.1 mean=1258.83 std=324.47 |
| Mean BM D (dose in bones Th1-Th10 inc. TVB) (Gy) | bm_md | **numerical** | min=2.42 max=21.71 median=9.31 mean=9.87 std=3.79 |
| V5BM (%) | bm_v05p | **numerical** | min=0.15 max=0.9 median=0.41 mean=0.43 std=0.13 |
| V10BM (%) | bm_v10p | **numerical** | min=0.08 max=0.68 median=0.3 mean=0.32 std=0.11 |
| V20BM (%) | bm_v20p | **numerical** | min=0.02 max=0.47 median=0.19 mean=0.19 std=0.09 |
| V30BM (%) | bm_v30p | **numerical** | min=0 max=0.32 median=0.08 mean=0.1 std=0.08 |
| V40BM (%) | bm_v40p | **numerical** | min=0 max=0.24 median=0.03 mean=0.05 std=0.05 |
| V PTV Volume planing target (cm³) | ptv_v | **numerical** | min=54.67 max=1149.2 median=297.62 mean=328.7 std=180.99 |
| Heart volume (cm³) | heart_v | **numerical** | min=369.5 max=1638.6 median=668.88 mean=717.91 std=204.43 |
| MHD Medium heart dose (Gy) | heart_mhd | **numerical** | min=0.08 max=24.54 median=6.24 mean=7.1 std=5.37 |
| V5H (%) | heart_v05p | **numerical** | min=0 max=1 median=0.31 mean=0.33 std=0.26 |
| V10H (%) | heart_v10p | **numerical** | min=0 max=0.9 median=0.17 mean=0.21 std=0.2 |
| Lung volume (cm³) | lung_v | **numerical** | min=1421 max=7665.11 median=3484.61 mean=3621.34 std=1069.61 |
| MLuD Medium lung dose (Gy) | lung_mld | **numerical** | min=2.89 max=22.8 median=13.25 mean=13.06 std=4.17 |
| V5Lu (%) | lung_v05p | **numerical** | min=0.12 max=1 median=0.58 mean=0.57 std=0.18 |
| V10Lu (%) | lung_v10p | **numerical** | min=0.02 max=0.77 median=0.39 mean=0.39 std=0.14 |
| MBD Medium body dose Th1-th10 (Gy) | mbd | **numerical** | min=1.24 max=19.87 median=9.06 mean=9.35 std=3.25 |
| CTCAE v5.0 (G0 >=1400/mm³,G1 <1400 - 800/mm³, G2 <800 - 500/mm³, G3 <500 - 200/mm³, G4 <200/mm3) | ctcae | **categorical** | G0(23)/G1(77)/G2(87)/G3(67)/G4(4) |
| Number of fraction | RT_fraction | **numerical** | min=15 max=34 median=30 mean=28.1 std=4.98 |
| EDIC | edic | **numerical** | min=1.68 max=9.43 median=4.59 mean=4.79 std=1.57 |
| ALC_TOX_G3G4 | ALC_TOX_G3G4 | **categorical** | 0(187)/1(71) |

**Supplementary Table S1.** Type, quantity of data, maximum(max), minimum(min), mean, median, standard deviation(std) are shown in Table S1.

**Characteristics of 30 best models~~.~~**

| **Id** | **Model** | **Features** | **No of features** | **MAE** | **MSE** | **RMSE** | **MAPE** | **Accuracy** |
| --- | --- | --- | --- | --- | --- | --- | --- | --- |
| 1 | Random Forest Regressor | alc_1, heart_v05p, lung_mld, lung_v05p | 4 | 0.213 | 0.076 | 0.274 | 39.52 | 60.48 |
| 2 | Random Forest Regressor | alc_1, lung_mld, lung_v05p | 3 | 0.215 | 0.076 | 0.274 | 39.53 | 60.47 |
| 3 | Random Forest Regressor | alc_1, lung_mld, lung_v05p, lung_v10p | 4 | 0.218 | 0.077 | 0.276 | 39.72 | 60.28 |
| 4 | ARD Regression | alc_1, lung_mld, lung_v05p | 3 | 0.219 | 0.078 | 0.276 | 39.94 | 60.06 |
| 5 | ARD Regression | alc_1, heart_v05p, lung_mld | 3 | 0.219 | 0.078 | 0.276 | 39.95 | 60.05 |
| 6 | ARD Regression | alc_1, lung_mld, lung_v05p, lung_v10p | 4 | 0.219 | 0.078 | 0.276 | 39.94 | 60.06 |
| 7 | ARD Regression | alc_1, heart_v05p, lung_mld, lung_v05p | 4 | 0.219 | 0.078 | 0.276 | 39.95 | 60.05 |
| 8 | Bayesian Ridge | alc_1, lung_mld, lung_v05p, lung_v10p | 4 | 0.219 | 0.078 | 0.277 | 39.87 | 60.13 |
| 9 | Bayesian Ridge | alc_1, lung_mld, lung_v05p | 3 | 0.219 | 0.078 | 0.277 | 39.90 | 60.10 |
| 10 | Linear Regression | alc_1, edic | 2 | 0.218 | 0.078 | 0.277 | 39.67 | 60.33 |
| 11 | Gaussian Process Regressor | alc_1, edic | 2 | 0.218 | 0.079 | 0.277 | 39.67 | 60.33 |
| 12 | Bayesian Ridge | alc_1, heart_v05p, lung_mld | 3 | 0.219 | 0.078 | 0.277 | 39.97 | 60.03 |
| 13 | Bayesian Ridge | alc_1, bm_v10p, ecog_ps, heart_v05p, lung_mld, lung_v10p | 6 | 0.217 | 0.078 | 0.277 | 39.46 | 60.54 |
| 14 | Linear Regression | alc_1, heart_v05p, lung_mld | 3 | 0.219 | 0.078 | 0.277 | 40.02 | 59.98 |
| 15 | Gaussian Process Regressor | alc_1, heart_v05p, lung_mld | 3 | 0.219 | 0.078 | 0.277 | 40.01 | 59.99 |
| 16 | Bayesian Ridge | alc_1, edic | 2 | 0.218 | 0.079 | 0.277 | 39.72 | 60.28 |
| 17 | ARD Regression | alc_1, edic | 2 | 0.219 | 0.079 | 0.277 | 39.83 | 60.17 |
| 18 | Bayesian Ridge | alc_1, heart_v05p, lung_mld, lung_v05p | 4 | 0.219 | 0.078 | 0.277 | 39.97 | 60.03 |
| 19 | Gaussian Process Regressor | alc_1, lung_mld, lung_v05p, lung_v10p | 4 | 0.219 | 0.078 | 0.277 | 39.70 | 60.30 |
| 20 | Gaussian Process Regressor | alc_1, lung_mld, lung_v05p | 3 | 0.219 | 0.079 | 0.277 | 39.97 | 60.03 |
| 21 | Linear Regression | alc_1, lung_mld, lung_v05p | 3 | 0.219 | 0.079 | 0.277 | 39.97 | 60.03 |
| 22 | ARD Regression | alc_1, bm_v10p, ecog_ps, heart_v05p, lung_mld, lung_v10p | 6 | 0.218 | 0.078 | 0.277 | 39.74 | 60.26 |
| 23 | Linear Regression | alc_1, lung_mld, lung_v05p, lung_v10p | 4 | 0.219 | 0.079 | 0.278 | 39.67 | 60.33 |
| 24 | ARD Regression | age, alc_1, bm_v30p, heart_mhd, heart_v10p, lung_mld, ptv_v, rt_duration, sex, vb_v05p | 10 | 0.222 | 0.078 | 0.278 | 40.51 | 59.49 |
| 25 | Gaussian Process Regressor | alc_1, heart_v05p, lung_mld, lung_v05p | 4 | 0.219 | 0.079 | 0.278 | 40.04 | 59.96 |
| 26 | Linear Regression | alc_1, heart_v05p, lung_mld, lung_v05p | 4 | 0.219 | 0.079 | 0.278 | 40.04 | 59.96 |
| 27 | ARD Regression | alc_1, lung_mld, vb_md, vb_v05p, vb_v10p, vb_v30p | 6 | 0.220 | 0.079 | 0.278 | 40.06 | 59.94 |
| 28 | Bayesian Ridge | alc_1, lung_mld, vb_md, vb_v05p, vb_v10p, vb_v30p | 6 | 0.220 | 0.079 | 0.278 | 40.15 | 59.85 |
| 29 | Linear Regression | alc_1, bm_md, bm_v05p, bm_v10p, bm_v20p, bm_v30p, bm_v40p | 7 | 0.224 | 0.079 | 0.278 | 40.96 | 59.04 |
| 30 | Huber Regressor | alc_1, edic | 2 | 0.217 | 0.079 | 0.278 | 38.16 | 61.84 |

**Supplementary Table S2.** Best 30 models according to Root Mean Square Error (RMSE) are shown.
